# Ileostomy Prolapse in Children with Intestinal Dysmotility

**DOI:** 10.1155/2017/7182429

**Published:** 2017-09-18

**Authors:** Eric A. Sparks, Cristine S. Velazco, Brenna S. Fullerton, Jeremy G. Fisher, Faraz A. Khan, Amber M. Hall, Tom Jaksic, Leonel Rodriguez, Biren P. Modi

**Affiliations:** ^1^Center for Advanced Intestinal Rehabilitation, Boston Children's Hospital, Boston, MA, USA; ^2^Department of Surgery, Boston Children's Hospital and Harvard Medical School, Boston, MA, USA; ^3^Department of Medicine, Division of Gastroenterology, Center for Gastrointestinal Motility and Functional Disorders, Boston Children's Hospital, Boston, MA, USA

## Abstract

**Background:**

A relationship between intestinal motility and ileostomy prolapse has been suggested but not demonstrated objectively.

**Aims:**

This study evaluated the association between ileostomy prolapse and intestinal dysmotility in children.

**Methods:**

IRB-approved retrospective review of 163 patients with ileostomies (1998–2014) at a single institution. Patients were categorized as having clinical dysmotility as a primary diagnosis (*n* = 33), clinically suspected dysmotility based on underlying diagnosis (*n* = 60), or intestinal dysmotility unlikely (*n* = 70) at the time of ileostomy present. Intestinal manometry was categorized as normal (*n* = 13) or abnormal (*n* = 10). Primary outcome was pathologic stoma prolapse. Multivariate analysis using a logistic regression model and log-rank test to compare stoma prolapse rates over time between motility groups were used.

**Results:**

Clinical diagnosis of dysmotility (*p* ≤ 0.001) and manometric findings of dysmotility (*p* = 0.024) were independently associated with stoma prolapse. Clinical dysmotility correlated with manometric findings (*κ* = 0.53). Prolapse occurred in 42% of patients with dysmotility, 34% of patients with suspected dysmotility, and 24% of patients with normal motility. One-year prolapse-free stoma “survival” was 45% for dysmotility, 72% for suspected dysmotility, and 85% for intestinal dysmotility unlikely groups (*p* = 0.006).

**Conclusions:**

Children with intestinal dysmotility are at great risk for stoma prolapse. Intestinal manometry could help identify these patients preoperatively.

## 1. Introduction

Chronic intestinal pseudo-obstruction (CIPO), first described in 1978, is a syndrome characterized by severe congenital gastrointestinal dysmotility and frequent episodes mimicking mechanical obstruction. Despite medical and surgical therapy, the morbidity and mortality associated with intestinal motility remain high [1, 2]. When surgery is required, both proximal (gastrostomy or jejunostomy) and distal (ileostomy or colostomy) decompression are frequently attempted [1]; however, these are not curative procedures and have high rates of complication [2, 3].

Ileostomy creation has been associated with frequent morbidities in both adults and children, including skin excoriation, bleeding, obstruction, stenosis, fistula formation, prolapse, malnutrition, and reoperations [4–6]. Prolapse is reported in 13% of adult ileostomies, and in the setting of CIPO, intestinal reoperations have been associated with increased mortality [3, 6]. A relationship between intestinal motility and ileostomy prolapse has been suggested anecdotally, yet this link has not been demonstrated objectively [7]. The purpose of this study is to evaluate the incidence of ileostomy prolapse in children with and without intestinal dysmotility.

## 2. Methods

Following IRB approval (protocol #P0012914), all patients with ileostomies present (1998–2014) at a single institution were identified using a systematic search of the electronic medical record for related diagnosis and ICD-9 codes (560.89, 560.8, 560.9, 564.8, 564.89, 564.9, 569.6, 569.60, 569.62, and 569.69) and CPT codes (44312, 44314, 4415, and 44310). After an original 205 patients were identified, 163 were verified by record review to have had ileostomies placed. Clinical characteristics recorded for each patient included indication for ileostomy creation, age, and weight at initial surgery, intestinal manometry results, and technical surgical details.

The hypothesis of this study was that patients with clinical and/or manometric small intestinal dysmotility would have a higher rate of ileostomy prolapse than patients with normal motility. To allow for a robust analysis, dysmotility was assessed with the use of both diagnostic categories based on clinical evaluation and intestinal manometry studies (*n* = 23). Prior to data analysis, each patient was reviewed by a gastroenterologist specializing in motility disorders to determine the best diagnostic category (L.R.). Patients with a clinical diagnosis of dysmotility or CIPO were placed in the “primary dysmotility” category (*n* = 33). Patients with a separate primary diagnosis but documentation of suspected dysmotility and patients with a diagnosis which frequently predisposes them to dysmotility (NEC, gastroschisis, atresia, aganglionosis, and cystic fibrosis) were categorized as “suspected dysmotility” (*n* = 60). All remaining patients were categorized as “intestinal dysmotility unlikely” (inflammatory bowel disease, constipation, anorectal malformations, and abdominal neoplasm; *n* = 70). Of note, patients with intractable constipation were included in the “intestinal dysmotility unlikely” category because they had no upper intestinal symptoms. All of these patients had their constipation symptoms resolved completely with placement of an ileostomy. This grouping scheme was validated by using Cohen's kappa coefficient to evaluate agreement between dysmotility categorization and the “gold-standard” diagnostic modality, intestinal manometry. Manometry studies were performed at the discretion of a gastroenterology staff both prior to and after initial ileostomy creation following a standardized protocol that includes 3 phases: fasting motility recording for 3 hours followed by feeding challenge when tolerated (either oral or via enteral tube) and a medication challenge (IV erythromycin to stimulate antral contractions and subcutaneous octreotide to stimulate phase III of the migratory motor complex).

The primary outcome was any pathologic stoma prolapse, defined as a prolapse episode requiring a physician-performed intervention (manual reduction with or without anesthesia, stoma revision, or stoma takedown) to achieve resolution. Patients were tracked until pathologic prolapse was documented, the stoma was reversed for other indications, or the patient was lost to follow-up. Demographic and clinical characteristics were assessed using Mann–Whitney *U* tests for continuous variables and Fisher's rank test for categorical variables. Multivariate analysis was performed by a logistic regression model for each possible predictor of prolapse. Variables with a *p* value ≤ 0.20 on univariate analysis were included in the final model. Kaplan-Meier survival tables and curves were generated and factors compared using the log-rank (Mantel-Cox) test.

## 3. Results

Clinical characteristics of the 163 patients with ileostomies included in this study are described in Table 1. At the time of ileostomy creation, these patients had a median age of 42 months (IQR 1.3–162). Median follow-up duration was 8 months (IQR 3–24). Most patients (76%) had end ileostomies while 9% had double-barrel ileostomies, and 15% had loop ileostomies.

The primary diagnosis requiring ileostomy creation for each patient is listed in Table 2. Of the total population, 33 patients had CIPO as a primary diagnosis and indication for ileostomy creation. 60 patients had dysmotility suspected based on either underlying diagnosis or clinical assessment. The remaining 70 patients had an anatomic or other indication for ileostomy creation unrelated to small bowel motility. 10 (43%) of 23 patients who underwent manometry testing were diagnosed as dysmotile. The dysmotility categories correlated well with manometric findings (*κ* = 0.53).

Overall, 38 patients (24%) had a documented episode of ileostomy prolapse requiring physician reduction or surgical revision at a median of only 2 months (IQR 0.3–3.5) following ileostomy creation. Sixty-eight (42%) patients underwent stoma takedown or revision prior to any prolapse after a median of 7.1 months (IQR 3.1–13.8). Fifty-six (34%) patients either reached the end of the study period without any prolapse (*n* = 10) or were lost to follow-up (*n* = 46) after a median of 30.6 months (IQR 14.5–62.8).

**Figure 1:**
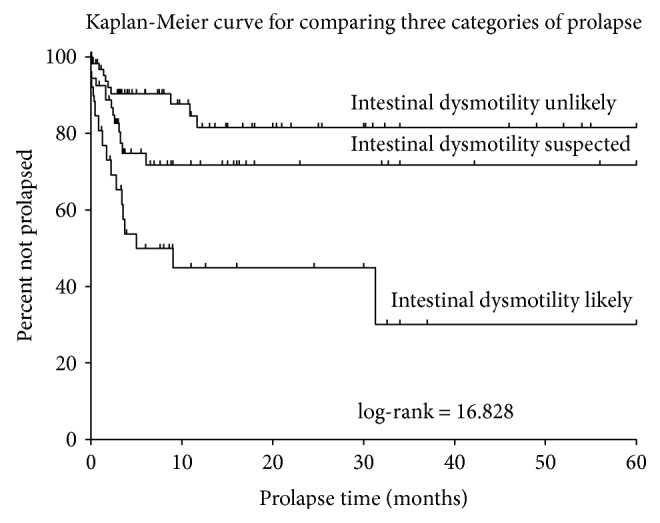
Predicted 5-year (60-month) prolapse for patients with an ileostomy by dysmotility category. Overall, a log-rank test determined that dysmotility category significantly differed with respect to the rate of prolapse (*χ*^2^ = 16.828; *p* < 0.001). Furthermore, a log-rank test established that confirmed dysmotility significantly differed from suspected dysmotility (*χ*^2^ = 5.386; *p* = 0.020) and unlikely dysmotility (*χ*^2^ = 16.926; *p* < 0.001); however, suspected dysmotility was not found to significantly differ from unlikely dysmotility (*χ*^2^ = 2.511; *p* = 0.11).

Prolapse occurred in 42% of patients with primary dysmotility, 34% of patients with clinically suspected dysmotility, and 24% of patients with intestinal dysmotility unlikely (*p* < 0.001). Following multivariate analysis (Table 3), only dysmotility confirmed by intestinal manometry remained significant (*p* = 0.031). No technical surgical variables (e.g., stoma location, laparoscopic versus open surgery, and fascial pexy) were associated with prolapse in this cohort. Log-rank testing estimated that one-year prolapse-free stoma “survival” was 45% for primary dysmotility, 72% for clinically suspected dysmotility, and 85% for dysmotility unlikely (*p* = 0.001, Figure 1).

## 4. Discussion

In this investigation, 163 children with end ileostomies were followed for a median of 8.4 (IQR 3–24) months to evaluate incidence of stoma prolapse, which was observed in 38 (24%) of all patients. Each patient was categorized into three groups based on clinical intestinal dysmotility, and this classification was validated by comparison to intestinal manometry results.

Multivariate regression demonstrated that intestinal manometry results diagnostic of dysmotility were associated with higher rates of prolapse. Significant agreement (*κ* = 0.53) was observed between manometry results and motility categorization, validating this approach for comparison. Prolapse appears to be primarily an early complication of ileostomy creation, occurring at a median of two months (IQR 0.3–3.5) and with only three occurrences of pathologic prolapse occurring more than one year after ileostomy creation. Consequently, the median follow-up duration of patients without prolapse was significantly longer than the median time to prolapse (*p* < 0.001). To further assess the possible interaction of dysmotility and prolapse, Kaplan-Meier analysis was performed. One-year prolapse-free stoma “survival” was poorest in patients with a primary diagnosis of intestinal dysmotility (*p* = 0.006).

Chronic intestinal pseudo-obstruction is described as an amalgam of congenital syndromes characterized by severe intestinal dysmotility with frequent symptoms of obstruction [8, 9]. Diagnosis is difficult and must follow careful elimination of other underlying pathologies including mechanical obstruction and aganglionosis [9, 10]. CIPO is frequently accompanied by abnormal biopsy or intestinal manometry results [10, 11]. Distinct variations of neurogenic and myogenic CIPO are reported, though many patients have idiopathic disease [11, 12]. Some series of familial disease have also been reported [13].

Successful medical and surgical management of children with CIPO has proven difficult, and multiple investigators have reported high rates of morbidity and mortality. The reported mortality rate varies between 0% and 23% [2, 3, 12, 14, 15]. Surviving patients frequently require long-term parenteral nutrition [1, 2, 12, 15] or eventual intestinal transplantation [1, 2]. Management is focused on improving nutrition and managing obstructive symptoms [1, 2, 16]. Surgery is generally limited to bowel decompression in the form of gastrostomy/jejunostomy and ileostomy or colostomy placement [1–3, 16, 17].

Ileostomy complications have frustrated patients and surgeons for as long as ileostomies have been performed [4, 6]. The morbidity of ostomies is especially high in infants and children [5, 18–20]. In particular, ileostomy prolapse has proven a frequent complication [7, 14, 21]. Many authors have suggested a variety of approaches to prevent or correct prolapse, including suture fixation of the bowel to the abdominal wall (stoma pexy), divided loop ileostomy, subcutaneous tunneling of bowel, Deflux® (hyaluronic acid/dextranomer, Salix Pharmaceuticals Inc., Raleigh, NC) injection, and stoma revision or takedown [21–29]. As none of these interventions have consistently proven effective, the potential risks and benefits of these options need to be very carefully assessed for each patient. This study was unable to identify any surgical technique which successfully reduced the incidence of stoma prolapse. Certainly, the lack of standardization in operative technique may contribute to the lack of any findings. In addition, it is possible, due to the retrospective nature of this analysis, that true differences could not be detected.

While intestinal motility correlated well with the clinical groups created, not all patients underwent upper intestinal manometry. The utility of preoperative manometry in identifying children at high risk for prolapse may be beneficial. Prospective investigations are necessary to determine if employment of this test may result in avoidance of an otherwise planned ileostomy or in more informed preoperative counseling of patients and families.

There are limitations to this study. Our study was retrospective in nature, and we cannot control for specific variations between surgeons for technical approach to ileostomy creation. Certainly, a prospective study controlling for surgical technique may determine if specific surgical techniques help prevent prolapse. Only 23 patients had manometry studies, limiting the number of patients diagnosed via this method. Prolapse may have influenced clinicians' decision making to diagnosis dysmotility for patients that were diagnosed postoperatively; in the future, preoperative manometry studies may help confirm dysmotility prior to stoma formation. Additionally, while this study did have a 25% loss to follow-up, this is not unexpected in a long-term retrospective analysis. Of note, the median observation period for patients lost to follow-up was 30.6 (IQR 14.0–63.4) months, which is longer than the median time to prolapse by an order of magnitude, suggesting that lack of follow-up in these patients is unlikely to have affected the outcome of the analysis.

These data corroborate findings by Irtan et al. who have suggested an association between CIPO and stoma prolapse in a small group of patients with ileostomies and colostomies [7]. Ileostomy prolapse appears to be a relatively frequent and early complication in patients with clinical and/or manometric dysmotility. Chronic intestinal pseudo-obstruction and other dysmotility syndromes result in the need for complex management, and patients undergoing ileostomy for decompression may see benefits which outweigh the potential morbidity of ileostomy prolapse. The knowledge that stoma prolapse is indeed seen with higher frequency in this patient population helps to inform surgical decision making and counseling and will hopefully result in more focused investigations such as evaluation of the increased use of manometry to predict ileostomy morbidity. Management by a multidisciplinary team (including pediatric surgery, gastroenterology, nutrition, social work, and transplant surgery) with experience in management of CIPO may be of benefit to limit high-risk procedures and aid future prospective investigation.

## Figures and Tables

**Table 1:** Population characteristics.

Descriptive and clinical variables	All patients
Age at stoma creation (months)	42 (1.2–163)
Gender, male	66 (41%)
Weight at stoma creation (kg)	14 (5–38)
WAZ score at stoma creation	−1.0 (−2.6 to −0.1)
On PN at stoma creation	64 (39%)
Surgical approach at stoma creation	
Laparoscopic	42 (29%)
Open	104 (71%)
Type of stoma	
End ileostomy	124 (76%)
Double-barrel ileostomy	15 (9%)
Loop ileostomy	24 (15%)
Surgical technique at stoma creation	
RLQ stoma placement	115 (71%)
Internal fascial pexy^∗^	1 (0.6%)
External fascial pexy^∗^	88 (54%)
Dysmotility Category	
Intestinal motility as primary diagnosis	33 (20%)
Intestinal dysmotility suspected	60 (37%)
Intestinal dysmotility unlikely	70 (43%)

Continuous variables are reported as median (IQR); frequencies are reported as *n* (%). WAZ: weight for age *z*-score; PN: parenteral nutrition; RLQ: right lower quadrant. ^∗^Operative data missing for 28 patients.

**Table 2:** Diagnosis leading to stoma creation in 163 patients with ileostomies, by motility category.

Diagnosis	*n*
Intestinal dysmotility as primary diagnosis	
Chronic intestinal pseudo-obstruction (CIPO)	33
Intestinal dysmotility suspected	
Necrotizing enterocolitis	30
Hirschsprung's disease	19
Cystic fibrosis	5
Enteric volvulus	2
Intestinal atresia	2
Gastroschisis	1
Meconium pseudocyst	1
Intestinal dysmotility unlikely	
Inflammatory bowel disease	36
Constipation	15
Anorectal malformations	7
Abdominal neoplasm	7
Others	5

**Table 3:** Descriptive and clinical variables for patients who did and did not experience pathological ileostomy prolapse.

	Prolapse	No prolapse	*p* value
Age at stoma creation (months)	29 (5–82)	55 (1–188)	0.31
Gender, male	15/38 (40%)	51/125 (41%)	0.88
Weight (kg) at stoma creation	13 (6–20)	16 (5–46)	0.12
WAZ score at stoma creation	−0.8 (−3.0 to −0.1)	−1.1 (−2.7 to −0.1)	0.45
Dysmotility by motility test (number of dysmotile/number tested)	7/10 (70%)	3/13 (23%)	0.024
On PN at stoma creation	15/38 (40%)	49/125 (39%)	0.98
Laparoscopic versus open stoma creation			0.23
Laparoscopic	7/34 (23%)	35/112 (28%)	
Open	27/34 (79%)	77/112 (62%)	
Type of stoma			0.4
End ileostomy	26/38 (68%)	98/125 (78%)	
Double-barrel ileostomy	4/38 (11%)	11/125 (9%)	
Loop ileostomy	8/38 (21%)	16/125 (13%)	
RLQ stoma placement	26/38 (68%)	89/125 (71%)	0.74
Internal stoma pexy^∗^	1/31 (3%)	0/104 (0%)	0.07
External fascia tacking^∗^	21/31 (68%)	67/104 (64%)	0.73
Dysmotility Category			<0.001
Intestinal motility as primary diagnosis	16/38 (42%)	17/125 (14%)	
Intestinal dysmotility suspected	13/38 (34%)	47/125 (38%)	
Intestinal dysmotility unlikely	9/38 (24%)	61/125 (49%)	

Continuous variables are reported as median (IQR); frequencies are reported as *n* (%). WAZ: weight for age *z*-score; PN: parenteral nutrition; RLQ: right lower quadrant. ^∗^Operative data missing for 7 patients in prolapse cohort and 21 patients in no prolapse cohort. Mann–Whitney *U* tests were used for continuous variables and chi-square tests for categorical variables.

## Abbreviations

CIPO: Chronic intestinal pseudo-obstruction.

## References

[1] Faulk D. L., Anuras S., Christensen J. (1978). Chronic intestinal pseudoobstruction. *Gastroenterology*.

[2] Lauro A., De Giorgio R., Pinna A. D. (2015). Advancement in the clinical management of intestinal pseudo-obstruction. *Expert Review of Gastroenterology & Hepatology*.

[3] Goulet O., Sauvat F., Jan D. (2005). Surgery for pediatric patients with chronic intestinal pseudo-obstruction syndrome. *Journal of Pediatric Gastroenterology and Nutrition*.

[4] Pakarinen M. P., Kurvinen A., Koivusalo A. I. (2013). Surgical treatment and outcomes of severe pediatric intestinal motility disorders requiring parenteral nutrition. *Journal of Pediatric Surgery*.

[5] Sabbagh C., Amiot A., Maggiori L., Corcos O., Joly F., Panis Y. (2013). Non-transplantation surgical approach for chronic intestinal pseudo-obstruction: analysis of 63 adult consecutive cases. *Neurogastroenterology and Motility*.

[6] Brooke B. N. (1952). The management of an ileostomy, including its complications. *Lancet*.

[7] Steinau G., Ruhl K. M., Hörnchen H., Schumpelick V. (2001). Enterostomy complications in infancy and childhood. *Langenbeck's Archives of Surgery*.

[8] Warren R., McKittrick L. S. (1951). Ileostomy for ulcerative colitis; technique, complications, and management. *Surgery, Gynecology & Obstetrics*.

[9] Irtan S., Bellaïche M., Brasher C., El Ghoneimi A., Cézard J. P., Bonnard A. (2010). Stomal prolapse in children with chronic intestinal pseudoobstruction: a frequent complication?. *Journal of Pediatric Surgery*.

[10] Goulet O., Jobert-Giraud A., Michel J. L. (1999). Chronic intestinal pseudo-obstruction syndrome in pediatric patients. *European Journal of Pediatric Surgery*.

[11] Cucchiara S., Annese V., Minella R. (1994). Antroduodenojejunal manometry in the diagnosis of chronic idiopathic intestinal pseudoobstruction in children. *Journal of Pediatric Gastroenterology and Nutrition*.

[12] Faure C., Goulet O., Ategbo S. (1999). Chronic intestinal pseudoobstruction syndrome: clinical analysis, outcome, and prognosis in 105 children. French-Speaking Group of Pediatric Gastroenterology. *Digestive Diseases and Sciences*.

[13] Shaw A., Shaffer H., Teja K., Kelly T., Grogan E., Bruni C. (1979). A perspective for pediatric surgeons: chronic idiopathic intestinal pseudoobstruction. *Journal of Pediatric Surgery*.

[14] Kim H.-Y., Kim J.-H., Jung S.-E., Lee S.-C., Park K.-W., Kim W.-K. (2005). Surgical treatment and prognosis of chronic intestinal pseudo-obstruction in children. *Journal of Pediatric Surgery*.

[15] Muto M., Matsufuji H., Tomomasa T. (2014). Pediatric chronic intestinal pseudo-obstruction is a rare, serious, and intractable disease: a report of a nationwide survey in Japan. *Journal of Pediatric Surgery*.

[16] Di Lorenzo C., Reddy S. N., Villanueva-Meyer J., Mena I., Martin S., Hyman P. E. (1991). Cisapride in children with chronic intestinal pseudoobstruction. An acute, double-blind, crossover, placebo-controlled trial. *Gastroenterology*.

[17] Murr M. M., Sarr M. G., Camilleri M. (1995). The surgeon’s role in the treatment of chronic intestinal pseudoobstruction. *The American Journal of Gastroenterology*.

[18] Musemeche C. A., Kosloske A. M., Ricketts R. R. (1987). Enterostomy in necrotizing enterocolitis: an analysis of techniques and timing of closure. *Journal of Pediatric Surgery*.

[19] O’Connor A., Sawin R. S. (1998). High morbidity of enterostomy and its closure in premature infants with necrotizing enterocolitis. *Archives of Surgery*.

[20] Weber T. R., Tracy T. F., Silen M. L., Powell M. A. (1995). Enterostomy and its closure in newborns. *Archives of Surgery*.

[21] Bielecki K. (2010). Recurrent ileostomy prolapse: is it a solved problem?. *Techniques in Coloproctology*.

[22] Canil K., Fitzgerald P., Lau G., Cameron G., Walton M. (1995). Button-pexy fixation for repair of ileostomy and colostomy prolapse. *Journal of Pediatric Surgery*.

[23] Crile G., Thomas C. Y. (1951). Skin graft for prolapse of ileostomy. *Surgery*.

[24] Ein S. H. (1984). Divided loop colostomy that does not prolapse. *American Journal of Surgery*.

[25] Festen C., Severijnen R. S., vd Staak F. H. (1988). Enterostomy complications in infants. *Acta Chirurgica Scandinavica*.

[26] Lichtenstein I. L., Herzikoff S. S. (1955). Recurrent ileostomy prolapse, an old problem; presenting a new approach. *Annals of Surgery*.

[27] Mayo C. (1939). Button colopexy for prolapse of colon through colonic stoma. *Staff Meetings of the Mayo Clinic*.

[28] Pomeranz A. A. (1963). An operation for prevention of ileostomy prolapse. *Diseases of the Colon and Rectum*.

[29] Sohn N., Schulman N., Weinstein M. A., Robbins R. D. (1983). Ileostomy prolapse repair utilizing bidirectional myotomy and a meshed split-thickness skin graft. *American Journal of Surgery*.

